# Development of Road Surface Detection Algorithm Using CycleGAN-Augmented Dataset

**DOI:** 10.3390/s21227769

**Published:** 2021-11-22

**Authors:** Wansik Choi, Jun Heo, Changsun Ahn

**Affiliations:** 1 School of Mechanical Engineering, Pusan National University, Busan 46241, Korea; cws8262@pusan.ac.kr; 2 Department of Mechanical Engineering, University of Michigan, Ann Arbor, MI 48109, USA; junheo@umich.edu

**Keywords:** deep neural network, CycleGAN, road surface detection, road friction detection

## Abstract

Road surface detection is important for safely driving autonomous vehicles. This is because the knowledge of road surface conditions, in particular, dry, wet, and snowy surfaces, should be considered for driving control of autonomous vehicles. With the rise of deep learning technology, road surface detection methods using deep neural networks (DNN) have been widely used for developing road surface detection algorithms. To apply DNN in road surface detection, the dataset should be large and well-balanced for accurate and robust performance. However, most of the images of road surfaces obtained through usual data collection processes are not well-balanced. Most of the collected surface images tend to be of dry surfaces because road surface conditions are highly correlated with weather conditions. This could be a challenge in developing road surface detection algorithms. This paper proposes a method to balance the imbalanced dataset using CycleGAN to improve the performance of a road surface detection algorithm. CycleGAN was used to artificially generate images of wet and snow-covered roads. The road surface detection algorithm trained using the CycleGAN-augmented dataset had a better IoU than the method using imbalanced basic datasets. This result shows that CycleGAN-generated images can be used as datasets for road surface detection to improve the performance of DNN, and this method can help make the data acquisition process easy.

## 1. Introduction

The knowledge of road surface conditions is one of the most important factors in safe autonomous driving. It is known that tire–road friction and road surface conditions are highly correlated with the rate of car crashes [1,2,3]. Detecting road surface types has been a popular research topic for several decades, and many corresponding projects are actively being conducted [4,5,6,7,8,9,10,11,12,13,14,15]. In the early days, many methods were developed using analytic approaches. For example, methods were developed by using reflected light to detect the road surface conditions [4], focusing on co-occurrence matrices [5], using a spatial filter [6], using polarization change, and through graininess analysis [7]. On the other hand, recently, many data-based methods have been developed for road surface detection with the rise of deep learning. For example, methods have been developed by using unsupervised learning [12], using the Convolutional Neural Network (CNN) with occupancy grid SVM [13], and applying CNN to classify an entire image as types of road conditions [14]. Currently, image semantic segmentation using deep learning seems to be the most popular method for road surface detection [16,17,18,19,20,21].

The deep learning-based methods show good performance when a well-balanced and sufficiently large dataset is used. If the dataset is imbalanced, the result will be biased. If the dataset is not sufficiently large, an overfitting problem is inevitable. However, acquiring large, balanced datasets requires a high-cost and time-consuming process. Although there are many datasets that are open to the public and contain road surface images, such as KITTI [22], Cityscapes [23], and Robocar [24], these datasets provide few wet and snowy road surface images, whereas dry road surface images are plentiful.

Imbalanced and small datasets are challenging for training any neural network, not only neural networks for surface detection algorithms. In research on the classification of objects in images, many interesting and effective methods have been developed to mitigate the problems caused by imbalanced and small datasets [25,26,27]. Among them, augmenting the dataset is the most popular and promising method.

In neural network-based road surface detection studies, most methodologies reviewed previously have been based on real data with sizes of several hundred to several thousand [12,13,14,15,16,17,18,19,20,21], which are expensive in time and cost. In this research, we propose a method that transforms dry road surface images into wet and snowy road surface images using Cycle-Consistent Adversarial Networks (CycleGAN), which can reduce the required number of images as well as the effort and time for data acquisition. This transformation technique can augment the dataset so that the dataset can be balanced with minimum cost. CycleGAN is an unsupervised learning method that converts images in a domain to images in another domain, such as a zebra to a horse, apples to oranges, and summer mountains to winter mountains, without data of paired images [28]. These artificially augmented data can improve the performance and robustness of neural network-based detection algorithms. To confirm the improvement, a DNN-based road surface detection algorithm was trained with the CycleGAN augmented dataset. This test result shows that the performance of the detection algorithm trained with the augmented dataset was better than that of the algorithm trained with the raw dataset. Therefore, the proposed method contributes to reducing the time and effort of data acquisition.

The rest of the paper consists of the following. Section 2 presents the data augmentation method. The validation of the proposed method and the discussion are in Section 3. Section 4 concludes the paper.

## 2. Dataset and Methods

**Figure 1:**
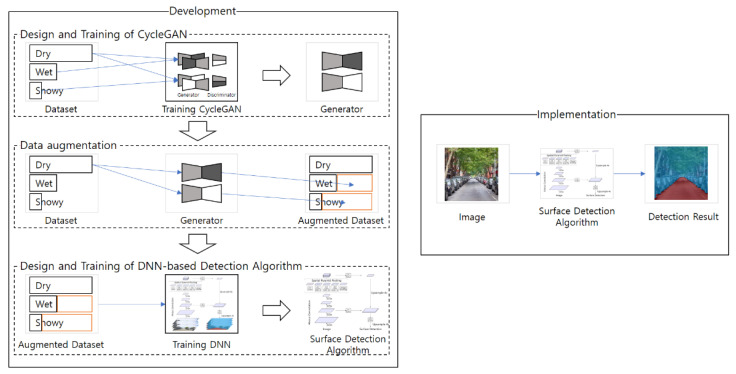
Steps of the proposed road surface detection method.

The proposed method to develop the road surface detection algorithm consists of three steps: the design and training of CycleGAN, data augmentation, and the design and training of the DNN for road surface detection, as shown in Figure 1. In the design and training of CycleGAN step, CycleGANs are trained to develop image translators (artificial image generators) for data augmentation. In the data augmentation step, the dry images are translated into wet and snowy images by the image translators. In the design and training of the DNN step, the augmented images are used for training the DNN-based road surface detection algorithm.

### 2.1. Base Dataset of Road Images

**Figure 2:**
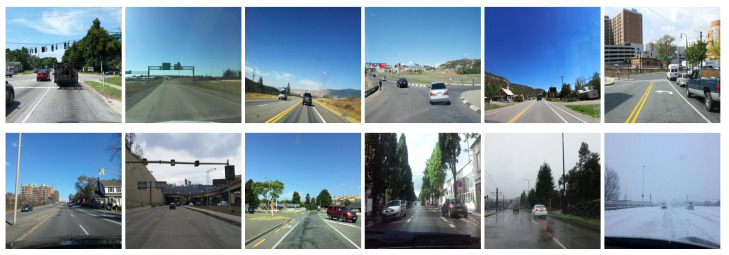
Sample images of Mapillary Vistas dataset.

We used Mapillary Vistas public dataset v1.1 [29] as a base dataset for road surface detection. The dataset contains 20,000 street-level images taken in different weather conditions, as shown in Figure 2.

**Figure 3:**
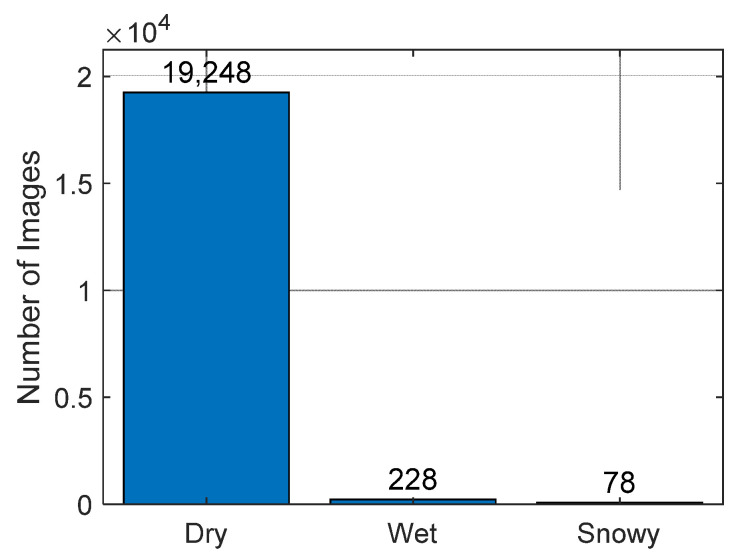
The number of images containing each road surface type in Mapillary Vistas dataset.

The dataset consists of three types of road images: 19,248 dry images, 228 wet images, and 78 snowy images, as shown in Figure 3.

### 2.2. Data Augmentation by CycleGAN

According to Figure 3, there is a huge imbalance between the number of dry, wet, and snowy road images. If a DNN is trained using the imbalanced data, overfitting will occur. To avoid this problem, the number of road images in each class should be large and well-balanced. However, acquiring well-balanced images is difficult because the weather conditions of the real environment during data acquisition are not balanced. One cost-efficient way to mitigate this problem is data augmentation.

To augment the data, we choose unsupervised learning, specifically, a generative network. The output of a generative network is trained to have similar stochastic characteristics to a specific dataset. The Generative Adversarial Network (GAN) is a famous generative network that has great performance [30]. GAN consists of two networks: a generator network and a discriminator network. In the GAN framework, the two networks have adversarial objectives. The generator produces fake data, whereas the discriminator distinguishes the fake data from the real data. The objective of training the discriminator is to accurately classify the fake and real data, and that of the generator is to deceive the discriminator. In this framework, the discriminator guides the training of the generator. With this process, the generator can be trained to generate data that have similar characteristics to the real dataset. For example, if GAN is trained using images with snowy road surfaces, the outputs of the generator would be images with snowy road surfaces.

Although GAN has great performance, applying the data augmentation technique to road surface detection is not simple. This is because GAN must be trained with the target dataset. For example, to generate snowy road images, GAN should be trained with a dataset of snowy road images. In addition, GAN should learn both images of the street view and the surface condition; therefore, a large dataset is required.

CycleGAN is an alternative method for data augmentation that does not require a large number of target images. CycleGAN is an image-to-image translation method based on GAN [28]. Unlike the other image-to-image translation methods, CycleGAN does not require paired training data. For example, general translation methods for road images require paired images taken from the same view with different road conditions. On the other hand, CycleGAN requires only a large number of dry road images and some snowy road images that are unpaired. Therefore, the number of images can be imbalanced for CycleGAN, which is a very useful feature for road surface data augmentation. To generate wet and snowy road images, many dry road images and small numbers of wet and snowy road images could be sufficient.

**Figure 4:**
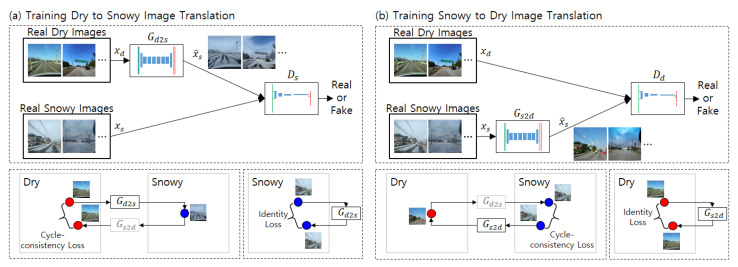
Concept of snowy road surface image data augmentation using CycleGAN.

CycleGAN has two pairs of generators and discriminators. The first pair translates an image in domain X into an image in domain Y. The second pair operates the other way around, translating an image in domain Y into an image in domain X. The loss function of the discriminator is the same as that of GAN. On the other hand, the loss function of the generator has two additional terms on top of the loss function of GAN: a cycle-consistency loss and an identity loss. The cycle-consistency loss is defined as follows. If an image in domain X is translated into domain Y and translated into domain X again (cycled translation), the ideal result should be that the original image and the image generated by cycled translation should be identical. Therefore, the cycle consistency loss is defined as the norm of the error between the original image and the image returned from cycled translation. Identity loss is defined as follows. If an image in domain X is translated into the same domain X, the results should be identical. Therefore, identity loss is defined as the norm of errors between the original images and translated images. The concepts of the losses are shown in Figure 4.

The overall structure of CycleGAN for road surface image translation is shown in Figure 4. In the figure, domain X is the snowy road surface, and domain Y is the dry road surface. Figure 4a shows the training structure for the dry to snowy image translation. The snowy image generator $G_{d2s}$ generates snowy images from real dry images. The snowy image discriminator $D_{s}$ classifies the real snowy images and the generated images. The classification loss is computed using the output of the discriminator. The cycle consistency loss is calculated by comparing the real dry images to the dry images generated through cycled translation. The identity loss is calculated by comparing the real snowy images to the snowy images translated into the same domain. $G_{d2s}$ is trained to maximize the classification loss and minimize both the cycle consistency loss and the identity loss. $D_{s}$ is trained to minimize the classification loss. Figure 4b shows the training for the snowy to dry translation, which is required to calculate the cycle consistency loss. The same method is applied for wet image data augmentation.

**Figure 5:**
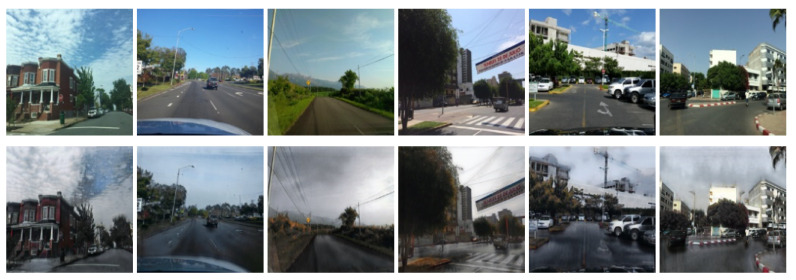
Sample images translated from dry conditions (**top images**) to wet conditions (**bottom images**).

**Figure 6:**
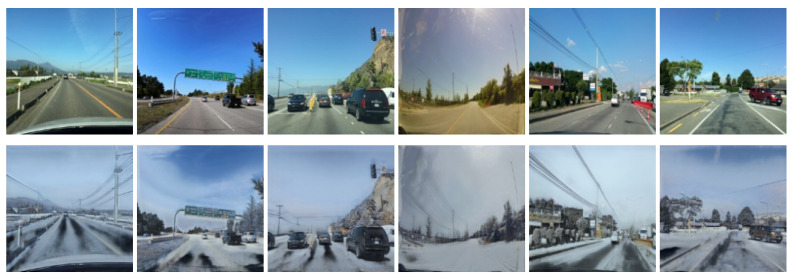
Sample images translated from dry conditions (**top images**) to snowy conditions (**bottom images**).

Figure 5 and Figure 6 show the results of the data augmentation for wet surfaces and snowy surfaces, respectively. For the augmentation, two CycleGANs (for wet surfaces and snowy surfaces) are trained with the base dataset. In both cases of augmentation, the sky is transformed to be cloudier than the original images, which is expected because wet or snowy surfaces are highly correlated with cloudy skies. In the wet condition case, the road surface images become darker than the original images, which is consistent with the usual observation that wet surfaces look darker than dry surfaces. In the snowy condition case, the road surface images are transformed to be covered by white snow.

### 2.3. Training Datasets for Road Surface Detection

**Figure 7:**
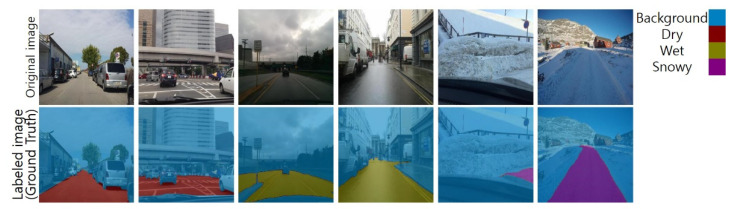
Sample images of the labeled images.

The road surface detection method classifies each pixel of road images as dry, wet, or snowy. We can interpret the detection as an image segmentation process. Therefore, a data-based image segmentation model was trained. To train the model, segments of road images in the dataset were labeled, as shown in Figure 7. The pixels were classified as four labels: dry, wet, snowy, and background. The background label means that the pixel is not of a road surface.

To train the road surface detection algorithm, two labeled datasets were used. One was the labeled dataset of original road images, which was called the baseline dataset, as shown in Table 1. The other was the labeled dataset of augmented road images, which was called the augmented dataset, as shown in Table 2. If the baseline dataset was used in the algorithm training, the result would have been highly biased because the number of wet and snowy images in the dataset is much smaller than that of dry surface images. Therefore, 500 dry images were selected out of 1000 labeled dry surface images for balanced training and testing. The number of wet surface images was 228, and that of snowy surface images was 78, and all were existing wet and snowy surface images in the original dataset. On the other hand, the augmented dataset contained 1000 dry surfaces images, 1228 wet surface images, and 1078 snowy surfaces images. Most wet and snowy surface images were artificially generated by CycleGAN.

### 2.4. Detection Algorithm

**Figure 8:**
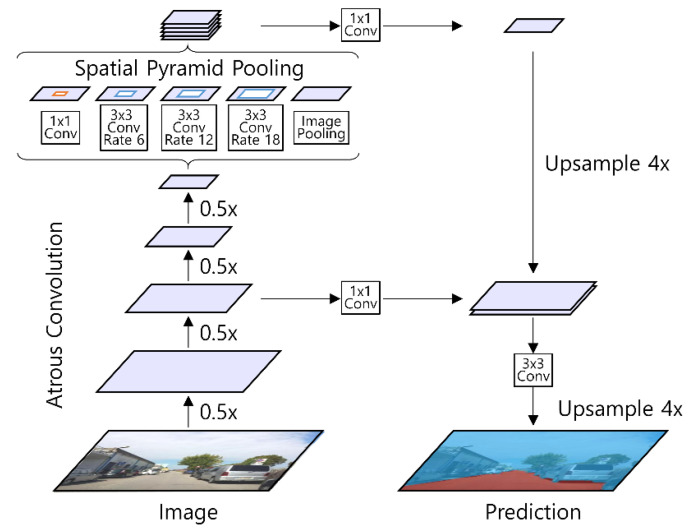
The structure of DeepLabv3+.

For road surface detection, we used the DeepLabv3+ model [31] shown in Figure 8. DeepLabv3+ is an extended model of DeepLabv3 that adds a simple and effective decoder module. This model shows excellent segmentation performance [31,32]. It can have a flexible area of the receptive field without increasing the number of parameters or the amount of calculation. The DeepLabv3+ model can conduct the segmentation process based on multi-scale context thanks to the atrous spatial pyramid pooling structure. The atrous spatial pyramid pooling structure concatenates outputs of atrous convolution with various rates and converts the concatenated images as an image using a 1 × 1 convolutional layer. The DeepLabv3+ model has a decoder with an intermediate connection similar to U-Net [33], which helps accurately predict the object boundary. The model requires a smaller number of parameters than the general convolution network by using depth-wise separable convolution.

Although the augmented dataset is balanced in the sense of the numbers of dry, wet, and snowy surface images, the dataset is still imbalanced in the sense of the numbers of pixels across all images. For example, more than half of the pixels in the images are classified as background. To further mitigate possible problems caused by the imbalanced dataset, we use median frequency balancing [27]. This method adopts weighting factors for each class when calculating the cross-entropy loss. The frequency of each class is used to calculate the weighting factors. The frequency is defined as the number of pixels of each class divided by the total number of pixels in the images that have pixels of that class. The weighting factors are the multiplicative inverses of the frequency divided by the median of the frequencies. Table 3 presents the weighting factors of each dataset. The loss function for an image with median frequency balancing is as follows:

(1) $$\mathrm{Loss}=-\sum_{i=1}^{N}w_{i}\sum_{j=1}^{M}y_{ij}\cdot \log{\hat{y}}_{ij},$$

where N is the number of pixels, i is the pixel index, $w_{i}$ is the weighting factor of the ith pixel, M is the number of classes, j is the class index, and $y_{ij}$ and ${\hat{y}}_{ij}$ are the label and output of the jth class and ith pixel.

## 3. Validation and Discussion

In this chapter, the results of the road surface detection algorithm are presented. For the purpose of comparison, two road surface detection algorithms are presented. One is an algorithm trained using the baseline dataset, which is called the baseline algorithm. The other is an algorithm trained using the augmented dataset, which is called the augmented algorithm.

**Figure 9:**
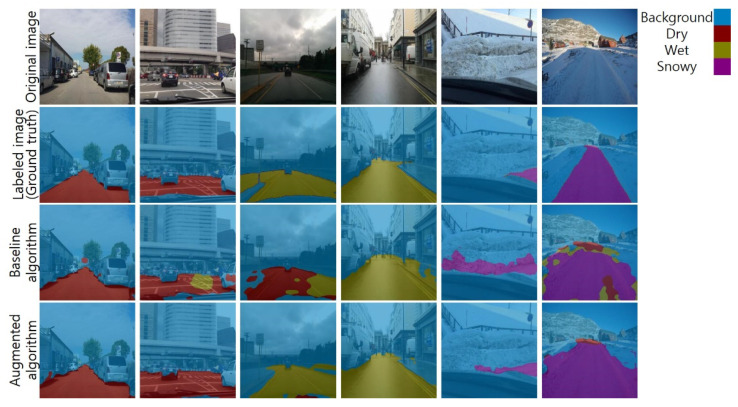
Road surface detection results.

Figure 9 shows selected results of road surface detection on two images of dry road, two images of wet road, and two images of snowy road. In the first column, both baseline and augmented algorithms show similar performance. However, other results show that the performance of the augmented algorithm is higher than that of the baseline algorithm. In the second and third columns, the baseline algorithm confuses dry surfaces and wet surfaces. In the fourth and fifth columns, the augmented algorithm shows more accurate road boundaries. The sixth column shows that both algorithms failed to detect the surface in some pixels; however, the augmented algorithm showed fewer failures. Qualitatively, the augmented algorithm showed superior performance to the baseline algorithm.

Table 4 shows the quantitative performance of each algorithm. The performance is measured using the precision, recall, accuracy, F1 score, and the mean of the intersection of union (IoU). As expected from the qualitative comparison in Figure 9, the quantitative comparison also confirmed that the augmented algorithm has a higher performance than the baseline algorithm.

**Figure 10:**
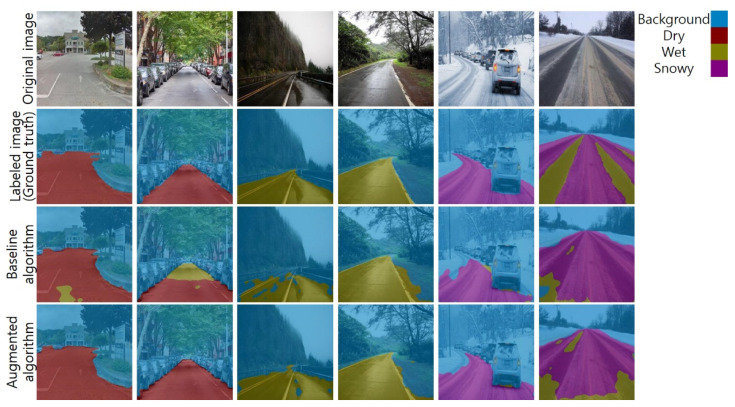
Road surface detection results using the new real images.

To strengthen the superiority of the augmented algorithm, the two algorithms were evaluated using a new test dataset. The new dataset consisted of only real road images taken in environments different from those in which the Mapillary Vistas dataset images were collected. We gathered 30 new road images. Ten of them contained dry surfaces, the other ten images contained wet surfaces, and others contained snowy surfaces. Figure 10 shows the selected results. Similar to the results in Figure 9, the performance of the augmented algorithm was higher than that of the baseline algorithm.

Table 5 shows the quantitative performance with the new images. Overall, the augmented algorithm showed superior performance to the baseline algorithm.

An interesting observation from Table 4 and Table 5 is that the IoU on the new real images was higher than that of the test set. This result was unexpected because, in general, additional data that did not affect training should have shown lower performance than the existing data. There could be two possible causes. The first one is that the number of new real images was too small. The significantly small number of the new real images could not generalize the performance evaluation. The second possibility is a human effect. When we gathered the new real images, we judged the class of each road image. In this procedure, ambiguous images were rejected; therefore, the results may be clearer than those of the test set.

## 4. Conclusions

For safe driving, drivers and vehicle control algorithms should consider the road surface conditions. DNNs can be a solution for this problem by being trained for road surface detection. However, the dataset is highly biased in general. Therefore, we introduced road surface detection trained with a CycleGAN-generated dataset. The suggested method showed better results compared with the baseline. In conclusion, road surface detection using the CycleGAN-generated dataset showed better results. The proposed approach can be applied to developing a classification algorithm with a small number of images and imbalanced datasets because of the cost and technical difficulties of artificially augmenting true-like data. The codes and data of the proposed method have been uploaded on Github (github.com/cws8262/Road_Surface_Detection_CycleGAN, accessed on 12 November 2021).

## Figures and Tables

**Table 1:** Baseline dataset.

	Dry	Wet	Snowy	Total
Training	300	137	47	484
Validation	100	46	16	162
Test	100	45	15	160
Total	500	228	78	806

**Table 2:** Augmented dataset.

	Dry	Wet	Snowy	Total
Training	600	737	647	1984
Validation	200	246	216	662
Test	200	245	215	660
Total	1000	1228	1078	3306

**Table 3:** Weighting factors for median frequency balancing.

	Baseline	Augmented
Background	0.2358	0.1785
Dry	0.9805	1.5894
Wet	1.0203	1.1374
Snowy	1.0818	0.8922

**Table 4:** Metrics of the test set trained with the baseline and augmented dataset.

	Baseline					Augmented				
	Precision	Recall	Accuracy	F1	IoU	Precision	Recall	Accuracy	F1	IoU
Background	0.84	0.95	0.77	0.89	0.93	0.95	0.94	0.89	0.94	0.93
Dry	0.93	0.91	0.84	0.92	0.79	0.91	0.96	0.87	0.94	0.80
Wet	0.89	0.87	0.76	0.88	0.66	0.90	0.91	0.81	0.90	0.73
Snowy	0.94	0.86	0.82	0.90	0.58	0.96	0.91	0.88	0.94	0.62
Total	0.90	0.90	0.80	0.90	0.74	0.93	0.93	0.86	0.93	0.77

**Table 5:** Metrics of the new real images trained with the baseline and augmented dataset.

	Baseline					Augmented				
	Precision	Recall	Accuracy	F1	IoU	Precision	Recall	Accuracy	F1	IoU
Background	0.81	0.94	0.72	0.87	0.90	0.89	0.92	0.81	0.90	0.90
Dry	0.97	0.91	0.89	0.94	0.81	0.96	0.99	0.96	0.98	0.84
Wet	0.88	0.86	0.75	0.87	0.71	0.97	0.85	0.82	0.91	0.77
Snowy	0.90	0.82	0.74	0.86	0.70	0.87	0.92	0.79	0.90	0.72
Total	0.89	0.89	0.77	0.89	0.78	0.92	0.92	0.84	0.92	0.81

## Data Availability

github.com/cws8262/Road_Surface_Detection_CycleGAN, accessed on 12 November 2021.
